# Citarinostat and Momelotinib co-target HDAC6 and JAK2/STAT3 in lymphoid malignant cell lines: a potential new therapeutic combination

**DOI:** 10.1007/s10495-020-01607-3

**Published:** 2020-05-11

**Authors:** Maria Cosenza, Monica Civallero, Luigi Marcheselli, Stefano Sacchi, Samantha Pozzi

**Affiliations:** 1 Unit of Oncohematology and Osteoncology, Department of Medical and Surgical Sciences, University of Modena and Reggio Emilia, Modena, Italy; 2 CHIMOMO Department, University of Modena and Reggio Emilia, Modena, Italy; 3 Fondazione Italiana Linfomi (FIL) onlus, University of Modena and Reggio Emilia, Modena, Italy

**Keywords:** Momelotinib, Citarinostat, HDAC inhibitor, JAK 1/2 inhibitor, Lymphoid malignancies, Synergistic combination

## Abstract

Histone deacetylase (HDAC) inhibitors represent an encouraging class of antitumor drugs. HDAC inhibitors induce a series of molecular and biological responses and minimal toxicity to normal cells. Citarinostat (Acy-241) is a second generation, orally administered, HDAC6-selective inhibitor. Momelotinib (CYT387) is an orally administered inhibitor of Janus kinase/signal transducer of transcription-3 (JAK/STAT3) signaling. Momelotinib showed efficacy in patients with myelofibrosis. We hypothesized that both HDAC and JAK/STAT pathways were important in lymphoproliferative disorders, and that inhibiting JAK/STAT3 and HDAC simultaneously might enhance the efficacy of momelotinib and citarinostat without increasing toxicity. Accordingly, we tested the citarinostat + momelotinib combination in lymphoid cell lines. Citarinostat + momelotinib showed strong cytotoxicity; it significantly reduced mitochondrial membrane potential, down-regulated Bcl-2 and Bcl-xL, and activated caspases 9 and 3. Caspase-8 was upregulated in only two lymphoid cell lines, which indicated activation of the extrinsic apoptotic pathway. We identified a lymphoid cell line that was only slightly sensitive to the combination treatment. We knocked down thioredoxin expression by transfecting with small interfering RNA that targeted thioredoxin. This knockdown increased cell sensitivity to the combination-induced cell death. The combination treatment reduced Bcl-2 expression, activated caspase 3, and significantly inhibited cell viability and clonogenic survival.

**Electronic supplementary material:**

The online version of this article (10.1007/s10495-020-01607-3) contains supplementary material, which is available to authorized users.

## Introduction

Histone deacetylases (HDACs) are master regulators of chromatin remodeling. HDACs can epigenetically control gene expression [1, 2], and they are considered promising therapeutic targets. Selective HDAC inhibitors (HDACis), alone or in combination with other anti-cancer agents, have shown encouraging results in cancer treatment strategies [3–6]. Recently, attention has focused on the HDAC6 isoform, due to its critical role in many biological functions. Through both deacetylase-dependent and -independent mechanisms, HDAC6 regulates numerous vital cell regulatory processes essential to normal and tumor cell growth, migration, and death [7–9].

Reports have shown that HDAC6 was overexpressed in lymphoid cells [10–12]. Agents that inhibit HDAC6 have demonstrated activity in preclinical and clinical studies [3, 4, 6, 13, 14]. Selective inhibition of HDAC6 might reduce the toxicity associated with off-target effects of pan-HDACis [7]. To that end, great effort has been dedicated to the search for selective HDAC6 inhibitors. Some inhibitors have shown strong HDAC6 selectivity; the development of these inhibitors could open up great prospects for applications related to cancer treatments [15]. Among the known HDAC6 inhibitors, only ricolinostat (rocilinostat, ACY-1215) and citarinostat (ACY-241) are currently under evaluation in clinical trials [16]. Ricolinostat is a first-in-class HDAC6 selective inhibitor. It exhibited acceptable tolerability, and preliminary studies have demonstrated its anti-myeloma efficacy, when given in combination with lenalidomide and dexamethasone. Additionally, pharmacodynamic evidence has shown that, in patients, ricolinostat could inhibit both HDAC6 and Class I HDACs. Citarinostat is a second generation, orally available, selective HDAC6 inhibitor [17]. It is structurally similar to ricolinostat, but it is administered as a tablet, rather than an oral solution. Compared to nonselective HDACis, citarinostat was well-tolerated, showed reduced potency against Class I HDACs, but had similar anticancer effectiveness [18].

Another potential therapeutic target for treating hematological malignancies is the Janus kinase (JAK) signaling pathway. JAKs are well described signaling kinases that comprise four family members: JAK1, JAK2, JAK3, and TYK2. JAKs are essential in hematological malignancies; indeed, JAK mutations were shown to contribute to the pathogenesis of myeloproliferative disorders [19, 20]. JAKs activate signal transducers of transcription (STATs), which, upon dimerization, migrate to the nucleus and induce the transcription of genes involved in the differentiation and proliferation of hematopoietic cells [20]. The JAK/STAT3 signal transduction pathway is downstream of cytokine receptors; it is activated in hematologic malignancies and various solid tumors [21]. Momelotinib (CYT387) is an orally administered drug that inhibits JAK1, JAK2, JAK3, and TYK2 kinases [22–24]. Momelotinib was an effective treatment in patients with primary and secondary myelofibrosis [25–27].

Based on these findings, together with the advantages of a double oral treatment, and the mild toxicity profiles of the single drugs, we tested the combination of citarinostat and momelotinib in lymphoid cell lines, as a potential therapeutic modality for lymphoid malignancies.

## Materials and methods

### Drugs and reagents

Citarinostat (Acy-241) was kindly provided by Acetylon Pharmaceuticals (Boston, Massachusetts, USA). Citarinostat is structurally related to ACY-1215, and it selectively inhibits HDAC6, with biological effects similar to those observed with ACY-1215. Momelotinib was purchased from Selleck Chemicals (Houston, TX, USA). Drugs were dissolved in 100% DMSO (Sigma Aldrich) to create 10^–2^ M stock solutions that were stored at − 80 °C. For use, these stock solutions were diluted with cell culture medium to the appropriate concentrations. In all experiments, the final concentration of DMSO (used as the vehicle) did not exceed 0.01%.

### Cell cultures

We used a panel of twelve lymphoid malignant cell lines, including: WSU-NHL, RL, Karpas-422 (follicular lymphoma—FL), Granta-519, Jeko-1 (mantle cell lymphoma—MCL), Hut-78 (cutaneous T cell lymphoma—CTCL), Karpas-299 (anaplastic large cell lymphoma—ALCL), L540, L1236 (Hodgkin’s lymphoma—HL), U266, RPMI8266 (multiple myeloma—MM), and MEC-1 (chronic lymphocytic leukemia—CLL). The Hut-78 cell line was purchased from the European Collection of Cell Cultures (ECACC). All other lymphoid cell lines were purchased from the German Collection of Microorganisms and Cell Cultures (DSMZ).

With the exception of the Granta-519 line, all lymphoid cell lines were cultured in RPMI-1640 supplemented with 10% fetal bovine serum (FBS), 2 mM glutamine, and 100 U/ml penicillin and streptomycin. For Granta-519 cells, DMEM was used in place of RPMI-1640. All cell lines used in this study were thawed from early passage stocks and were passaged for less than 6 months.

Human mesenchymal stem cells (hMSCs) were purchased from Tebu Bio (UK). Cells in the logarithm growth phase were used for experiments.

Primary lymphoma cells were obtained from bone marrow (BM) samples from two patients with FL, three patients with MCL, and one patient with CTCL. Blood samples were drawn from three healthy volunteers, and peripheral blood mononuclear cells (PBMCs) were isolated with the Ficoll–Hypaque technique.

The study protocol was approved by the local Institutional Review Board. Written informed consent was obtained before sample collection. All reagents were purchased from Euroclone (Italy).

### Viability assay and analysis of synergism

Cell viability was evaluated with an MTS colorimetric assay (CellTiter, non-radioactive cell proliferation assay, Promega Corporation), according to manufacturer instructions, and with an exclusion assay, with 0.2% Trypan Blue (Euroclone).

Lymphoid cell lines were incubated in triplicate with increasing concentrations of either citarinostat (1 – 100 μM) or momelotinib (1—10 μM) for 24 – 48 h to identify the IC_50_ values for each drug. The IC_50_ values were used to determine the fixed ratio for the two agents in combination. Drug dilutions and combinations were prepared in media immediately prior to use. To assess drug combination effects, serial dilutions of the two agents were prepared at lower concentrations than the IC_50_, and lymphoid cell lines were cultured for 24 and 48 h. The experiments were conducted with the following concentrations of citarinostat: 0, 0.5, 1, 2, and 4 µM, combined with the following concentrations of momelotinib: 0, 0.125, 0.25, 0.5, and 1 μM. To investigate the inhibitory effects of the drugs and their combinations, we performed an isobologram analysis according to the Chou-Talalay method. This method provided algorithms for automated computer simulations of synergism and/or antagonism, based on the median-effect equation derived from the mass action law [28, 29]. Combination indices (CIs) were calculated for a 50% cell kill, according to the method of Chou and Talalay, where CI values < 1, = 1, and > 1 indicated synergy, additivity, and antagonism, respectively. We performed dose–effect analyses and synergism/antagonism quantifications with Calcusyn Software (Biosoft, Cambridge, UK).

### Lymphoid cell lines co-cultured with human mesenchymal stem cells

Mesenchymal stem cells (MSCs) were cultured according to the recommended protocol. MSCs were seeded in triplicate onto 96-wells plates, and incubated for 48 h to confluence. After 48 h, lymphoma cell lines were seeded in the presence or absence of MSCs. The next day, cells were treated with citarinostat, alone or in combination with momelotinib. Non-adherent cells were collected at 24, 48, and 72 h after adding the drugs. Cell viability was evaluated with the MTS assay.

### Clonogenic assay

Lymphoid cell lines were first exposed to citarinostat or momelotinib, alone or in combination, in liquid cultures for 24 – 48 h, or 6 h after cells were transfected with small interfering RNAs (siRNAs). Then, lymphoid cells were collected and incubated in methylcellulose media (Stemcell Technologies) and maintained for 14 days at 37 °C. Growing colonies (> 50 cells) were counted under a microscope.

### Migration assays

Migration was determined with Millicell Cell Culture Inserts (Millipore, Billerica, USA). Lymphoid cells were treated for 90 min with 4 μM citarinostat and/or 1 μM momelotinib or vehicle. Briefly, 5 × 10^5^ cells, resuspended in 200 µL RPMI-1640 medium with 0.5% bovine serum albumin (BSA, Sigma-Aldrich, St. Luis, MO, USA), were added to the upper compartment of the chamber. Then, an optimal concentration (200 ng/mL) of the chemokine, CXCL12 (also known as stromal-cell derived factor-1, SDF-1α; Peprotech, Rocky Hill, NJ, USA), was added to the lower chamber in 600 µL of the same medium that was used in the top chamber. The chambers were incubated at 37 °C in humidified air with 5% CO_2_ for 24 h. After 24 h, migrated cells were recovered from the lower chamber and counted with flow cytometry for 60 s, after calibrating the flow rate. To quantify cell migration, we applied the following formula: %migration = 100% × migrated cells/initial number of cells. Each experiment was performed in duplicate.

### Cell cycle analysis and apoptosis assay

Cell cycle analysis was determined by flow cytometry, as described previously [6]. We performed flow cytometry (FACS Calibur, BD) and used Cell Quest data analysis software to estimate the percentages of cells in the following phases of the cell cycle: sub G1/G0 (dead cells), G1/G0, S, and G2/M. Apoptosis was quantified with the Annexin V-FITC apoptosis detection kit (eBioscience, San Diego, CA, USA), according to the manufacturer’s instructions. Cells were analyzed with flow cytometry and Cell Quest data analysis software. Apoptotic cells were designated as early (Annexin V + /PI −) or late (Annexin V + /PI +) apoptosis.

### Caspase activity assay

The activities of caspases 3, 8, and 9 were determined with caspase-3, -8, and -9 colorimetric assay kits (R&D System, Minneapolis, USA), according to the supplier’s manual. Briefly, cells were resuspended in cell lysis buffer and centrifuged. Supernatants were collected to determine protein concentrations. Then, samples were incubated at 37 °C with reaction buffer and substrate for 1–2 h. Caspase expression levels were evaluated after 1 h of pretreatment with 40 μM of the broad caspase inhibitor, zVAD-fmk (Sigma-Aldrich, St. Luis, MO, USA). Samples were analyzed with a microplate reader (Infinite M200, Tecan, Männedorf, Switzerland).

### Analysis of mitochondrial membrane potential

The mitochondrial membrane potential (MMP) was measured with the JC-1 Mitochondrial Membrane Potential Assay Kit (Abcam, Cambridge, UK), according to manufacturer instructions. JC-1 is a fluorescent carbocyanine dye that accumulates in the mitochondrial membrane as a monomer or dimer, depending on the mitochondrial membrane potential. The fluorescence intensity of each sample was measured with a fluorescent plate reader (excitation: 475 nm; emission: 590 nm, for JC-1 monomers and JC-1 aggregates). The MMP in each group was calculated as the ratio of red to green fluorescence, and it was expressed as a percentage of the vehicle control. For the positive control, cells were incubated with FCCP (carbonyl cyanide 4-(trifluoromethoxy) phenylhydrazone), before adding the JC-1 solution. All experiments were performed in triplicate. The results are expressed as the mean ± SD.

### Measurement of cytochrome c release

The cytosolic cytochrome c content in lymphoid cells was quantified with a commercially available ELISA kit, according to manufacturer instructions (R&D Systems, Minneapolis, USA).

### Assessment of reactive oxygen species with flow cytometry

Cells were treated for 24 h, then incubated with 5 μM 2′,7′-dichloroflourescein diacetate (DCFH-DA; Sigma-Aldrich St. Louis, MO, USA) in PBS at 37 °C for 30 min. We used the free radical scavenger, N-acetyl-l-cysteine (NAC; Sigma-Aldrich St. Louis, MO, USA) to assess whether the generation of reactive oxygen species (ROS) played a role in apoptosis. Cells were pre-incubated with 12 mM NAC for 3 h, followed by incubation with citarinostat and momelotinib, either alone or in combination. H_2_O_2_ was used as a positive control. The fluorescence intensity was read with flow cytometry on the FL1 channel within 45 min. ROS production was determined in gated live cells by comparing fluorescence intensities in treated versus untreated cells. The data were analyzed with Cell Quest data analysis software.

### ATP assay and lactate

We measured ATP levels with an ATP assay colorimetric kit (Abcam, Cambridge, UK) according to manufacturer instructions. The ATP assay kit was based on the phosphorylation of glycerol, which generated a product that could be colorimetrically quantified (OD = 570 nm). Concentrations of lactate were measured with an L-lactate assay colorimetric kit (Abcam, Cambridge, UK), according to manufacturer instructions. Lactate was oxidized by lactate dehydrogenase to generate a product that interacted with a probe, which produced a color (OD = 450 nm). Absorbance readings were performed in a microplate reader (Infinite M200, Tecan, Männedorf, Switzerland).

### Western blot analysis and signaling assays

Cultured cells were harvested and resuspended in cold lysis buffer (Mammalian Cell Extraction Kit; Biovision Inc. CA, USA) according to manufacturer instructions. Cell lysates (50–100 μg protein) were loaded onto 4–20% (w/v) Miniprotean TGX Precast Gels (Biorad laboratories, Hercules, CA, USA), subjected to electrophoresis, and electrotransferred onto nitrocellulose membranes (Biorad Laboratories). The membranes were incubated overnight at 4 °C with antibodies specific for the following proteins: Jak, phosphorylated-Jak2 (p-Jak2), Stat3, p-Stat3, Stat5, p-Stat5, p21, p27, cyclin D, Bip, CHOP, PERK, p-PERK, thioredoxin 1, Bax, Bim, BCL-xL, PARP, acetyl-alpha, and tubulin. Next, membranes were incubated with species-specific horseradish peroxidase (HRP)-conjugated secondary antibodies (Bethyl Laboratories, Montgomery, TX USA) for 1 h, then developed with HRP substrate from Western Bright Sirius (Advansta, Menlo Park, CA, USA). The majority of antibodies were purchased from Cell Signaling Technology (Beverly, MA, USA). Images were acquired and analyzed with Image Lab Software v.3.0 (Biorad Laboratories).

### siRNA transfection

Granta-519 cells were transfected with an Amaxa 4D Nucleofector (Lonza, Cologne, Germany). Briefly, 2.5 × 10^6^ cells were resuspended in SF solution (Lonza, Cologne, Germany). A mixture of three different siRNA oligonucleotides directed against Trx (Trx1, Trx2, and Trx3; IDT, Coralville, Iowa, USA) was added to the cell solution. According to the protocol, we used transfection program DN113. In each reaction, a scrambled sequence and a green fluorescent protein (GFP) vector (2 µg) were used as controls. Transfected cells were incubated for 24 h before the indicated treatment. At 48 h after transfection, cells were collected for a viability assay and for immunoblotting. For the clonogenic test, Granta-519 cells were seeded at 6 h after transfection and assayed as described above. Transfection efficiency was measured with flow cytometry by detecting cells that expressed GFP. For Granta-519 cells, the transfection efficiency was ~ 68%.

### Statistical analysis, isobologram, and combination index calculation

All in vitro experiments were performed in triplicate, and repeated at least three times. Representative experiments were selected for the figures. Data are expressed as the mean ± standard deviation (SD). Statistical differences between controls and drug-treated cells were determined with a one-way analysis of variance (ANOVA). P-values < 0.05 were considered statistically significant. Data were analyzed with the Stata 8.2/SE package (StataCorp LP).

## Results

### Synergistic effects of citarinostat + momelotinib on cytotoxicity in malignant hematological cell lines

**Fig. 1:**
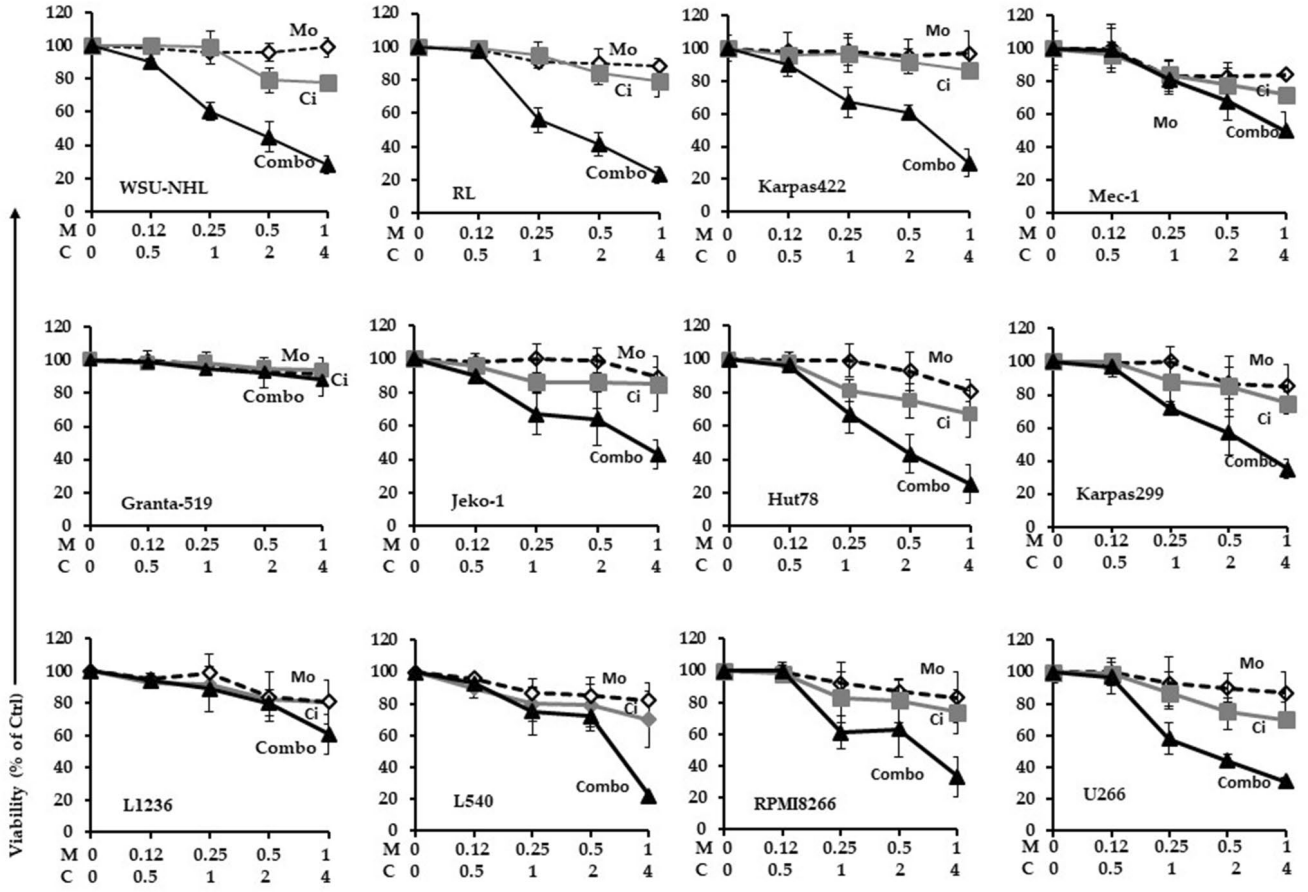
A panel of 12 lymphoid cell lines were treated with different concentrations of citarinostat (C = 0, 0.5, 1, 2, 4 μM) in combination with momelotinib (M = 0, 0.12, 0.25, 0.5, 1 μM). A synergistic interaction was observed at 24 h with citarinostat to 4 μM and momelotinib to 1 μM in WSU-NHL, RL, Karpas-422, Jeko-1, Hut-78, Karpas-299, L-540, RPMI8226 and U266 cells and Mec-1 with a combination index (CI) < 1. The antagonist effect was observed in L-1236, Granta-519 cells with CI > 1. Results represent the mean ± SD obtained from three independent experiment

**Fig. 2:**
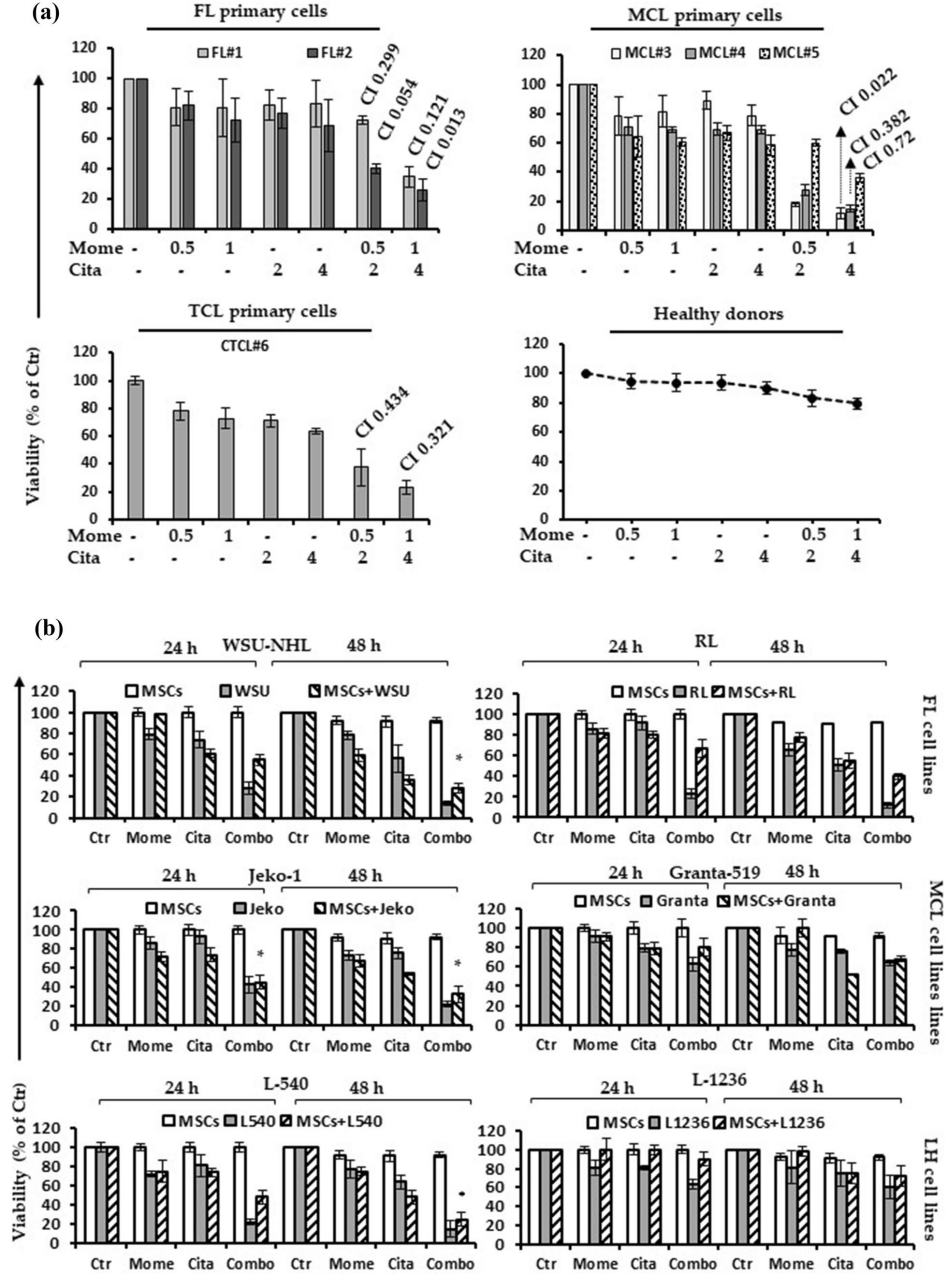
**a** Primary cells were isolated from two FL patients (FL#1, FL#2), three MCL patients (MCL#3, MCL#4 and MCL#5), one CTCL (CTLC#6) patient and three healthy subjects. Viability was evaluated with MTS assay. The synergistic effect is confirmed with the isobologram analysis. Combination indices (CI) are reported in each graph. Data are expressed as a percentage of untreated control cells and represent the mean ± SD of triplicate culture. **b** Cell viability of four lymphoid cell lines sensitive to the drug combination (CI < 1) (WSU, RL, Jeko-1 and L540) and two less sensitive lymphoid cell line (CI > 1) (Granta-519 and L-1236) co-cultured with or without BM-MSCs for 24 – 48 h. (*p < 0.001 statistically significant differences vs and single agents)

We first defined the cytotoxic activity of citarinostat and momelotinib as single agents. We cultured twelve cell lines in the presence or absence of citarinostat (0 to 50 µM) or momelotinib (0 to 10 µM) for 24 – 48 h. We performed the MTS assay to quantify cell viability. Exposure to citarinostat alone for 24—48 h resulted in a time- and dose-dependent inhibition of cell growth; IC_50_ values ranged from 4 to 50 μM (Fig. s1). A significant cytotoxic effect was observed after 48 h of treatment. Exposure to momelotinib alone resulted in anti-proliferation activity; IC_50_ values ranged from 1.8 to 9.6 μM (Fig. s2). To determine how cell viability was influenced by the citarinostat + momelotinib combination, we incubated all cell lines for 24 h with both drugs at different concentration ratios (1:0.25, 1:0.5, and 1:1). The goals of the combination treatment were to achieve synergistic therapeutic effects, reduce the effective dose and toxicity, and minimize or delay the induction of drug resistance [30]. Combination studies were performed at 24 h before extensive apoptosis started. We used concentrations below the IC_50_ values of the respective drugs. All lymphoid cell lines were treated with increasing concentrations of citarinostat (0, 0.5, 1, 2, and 4 μM) alone or in combination with momelotinib (0, 0.12, 0.25, 0.5, and 1 μM). Cell viability was assayed with MTS. The toxicity of each treatment was assessed by measuring the rate of growth inhibition. After 24-h exposures to citarinostat + momelotinib combinations, we observed an antagonistic effect (CI > 1) in Granta-519 and L-1236 cell lines. In the remaining cell lines (WSU-NHL, RL, Karpas-422, Jeko-1, Hut-78, Karpas-299, L-540, RPMI8226, U266, and Mec-1), the combination treatment inhibited cell viability and induced strong cytotoxic effects. CIs for the combination of 4 µM citarinostat and 1 µM momelotinib ranged from 0.02 to 0.60 (Table 1 and Fig. 1). We also established the synergistic activity of the combination treatment in primary cells isolated from blood samples of patients with lymphoma. We found that combinations of citarinostat (2 and 4 µM) and momelotinib (0.5 and 1 µM) exerted strong synergy in all the samples studied (n = 5). The CIs for the combination of 2 µM citarinostat and 0.5 µM momelotinib ranged from 0.022 to 0.38. The CIs for the combination of 4 µM citarinostat and 1 µM momelotinib ranged from 0.013 to 0.72 (Fig. 2a). Importantly, the combination treatment had minimal or no cytotoxic effect on PBMCs from healthy donors (Fig. 2a, bottom right).

**Table 1:** Analysis of drug combination effects

Citarinostat (µm)	Momelotinib (µM)	CI (CI 95%)
WSU-NHL		
2	0.5	0.30 (0.06–1.39)
4	1	0.45 (0.06–2.77)
RL		
2	0.5	0.14 (0.03–0.28)
4	1	0.13 (0.02–0.91)
KARPAS422		
2	0.5	0.34 (0.23–0.49)
4	1	0.13 (0.17–0.42)
MEC1		
2	0.5	0.35 (0.28–0.43)
4	1	0.16 (0.12–0.22)
GRANTA519		
2	0.5	1.21 (0.15–1.66)
4	1	1.76 (0.35–3.93)
JEKO1		
2	0.5	0.09 (0.02–0.36)
4	1	0.09 (0.02–0.55)
HUT78		
2	0.5	0.19 (0.04–0.89)
4	1	0.16 (0.02–1.68)
KARPAS299		
2	0.5	0.98 (0.16–1.71)
4	1	0.52 (0.14–2.52)
L1236		
2	0.5	1.21 (0.36–4.04)
4	1	1.17 (0.37–3.73)
L540		
2	0.5	0.57 (0.27–1.22)
4	1	0.05 ( 0 − 0.74)
RPMI8226		
2	0.5	0.16 (0.04–0.63)
4	1	0.02 (0.01–0.34)
U266		
2	0.5	0.18 (0.08–0.40)
4	1	0.17 (0.04–0.80)

Combination Index (CI) of citarinostat (2, 4 µM) in combination with momelotinib (0.5, 1 µM) after 24 h of treatment. Synergism, additivity, or antagonism were quantified by determining the CI calculated by the Chou–Talalay equation. Combination index (CI) CI < 1, synergism; CI = 1, additive effect; CI > 1, antagonism

Based on these results, we selected five lymphoid cell lines (WSU-NHL, Karpas-422, RL, Jeko-1, L-540) that were particularly sensitive to the drug combination (CIs < 1) and two cell lines (L-1236, Granta-519) with ICs > 1. For all further analyses, we treated cells with 4 μM citarinostat + 1 μM momelotinib. These doses were below the IC_50_ values of both drugs, and according to the Chou-Talalay method, they displayed strong synergy (CI < 1).

### Citarinostat + momelotinib maintained cytotoxicity in a bone marrow microenvironment

The bone marrow microenvironment can confer growth benefits and induce drug resistance in malignant cells [31]. Therefore, we performed a co-culture assay to examine the potential protective effect of MSCs on lymphoid cell lines. All selected lymphoid cell lines were cultured with 4 µM citarinostat and 1 µM momelotinib, alone or in combination, in the presence or absence of hMSCs for 24, 48, and 72 h. Citarinostat and momelotinib alone and in combination exerted minimal or no cytotoxic effect on stromal cells. A 24-h treatment with the drug combination caused insignificant cytotoxicity in Granta-519 and L-1236 cells, but it significantly reduced cell viability in the other five sensitive lymphoid cell lines. These results indicated that the treatment overcame the protective effects conferred by the bone marrow microenvironment (Fig. 2b). The synergistic effects of the citarinostat + momelotinib combination significantly increased after 48 h of co-culture. This result confirmed the that drug combination improved the cytotoxic effects of each drug alone, even in presence of hMSCs (Fig. 2b).

### Drug combination inhibits lymphoid cell colony formation and migration

**Fig. 3:**
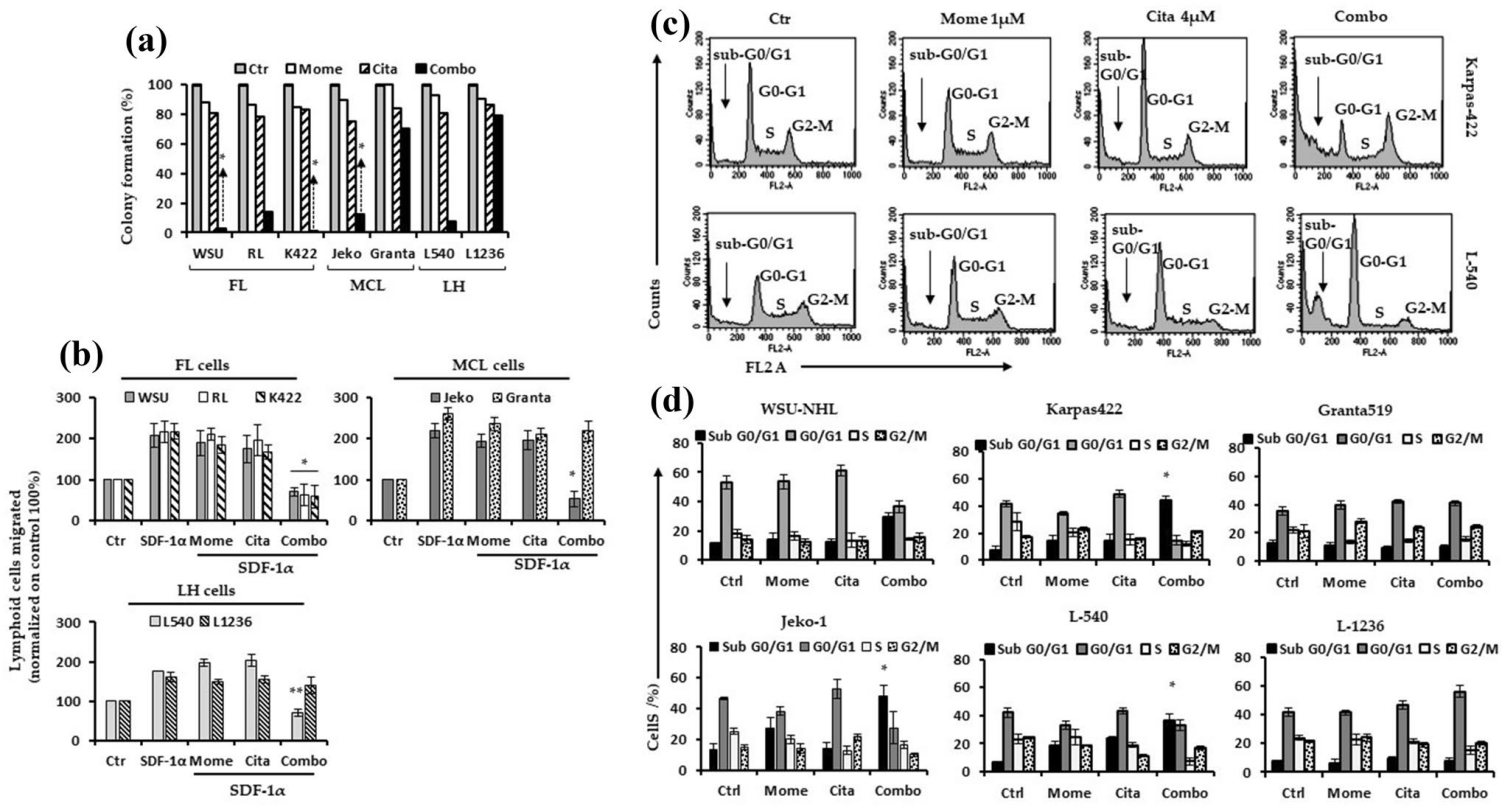
**a** Five lymphoid cell lines particularly sensitive to the drug combination with CI < 1 (WSU, Karpas422, RL, Jeko-1 and L540) and two less sensitive lymphoid cells with CI > 1 (Granta519 and L-1236) were treated with citarinostat (4 µM) and momelotinib (1 µM) alone and in combination in liquid culture for 24 h. After treatment, cells were incubated in methylcellulose. The relative percentage with respect to control cells are shown and represent the mean ± SD of three separate experiments (*p < 0.001 vs drugs alone). **b** Drug combination inhibited the migration induced by chemoattractant SDF-1α compared with control with no SDF-1α. SDF-1α was placed in the lower chambers of the drug combination-treated wells. **c** Representative flow cytometry histogram of the cell cycle distribution of the Karpas-422 and L-540 cell lines. **d** Cell cycle distribution (%) of four cell lines sensitive to the combination (WSU-NHL, Karpas422, RL and L-540) and two lines with CI > 1 (Granta-519 and L-1236) after 24 h of treatment. Drug combination induced an increase of sub G0/G1 phase in all the four cell lines. Values represent the mean ± SD of three independent experiments. (* p < 0.001)

We performed a clonogenic assay to study the effects of the drug combination on cell repopulating capacity. All lymphoid cells were incubated with 4 µM citarinostat and 1 µM momelotinib, alone or in combination, for 24 h. Next, cells were washed and seeded in semisolid media. In sensitive lymphoid cells, this clonogenic assay revealed that, compared to the single drug treatment, the combination treatment significantly inhibited colony formation by 86–90% (Fig. 3a). In contrast, Granta-519 and L-1236 cells were less sensitive to treatment; after 24 h treatments, 25% of Granta-519 cells and 20% of L-1236 cells displayed clonogenic survival (Fig. 3a). We performed a cell migration assay to evaluate the effects of the combination treatment on lymphoid cell migratory capacity. Lymphoid cells were incubated with citarinostat (4 µM), either alone or in combination with momelotinib (1 µM) for 90 min. We confirmed that these doses and incubation times did not cause a reduction in cell viability with MTS and trypan blue assays (data not shown). We then examined cell migration. As shown in Fig. 3b, the treatment with citarinostat or momelotinib alone did not inhibit cell migration induced by CXCL12 (a chemoattractant). In contrast, the drug combination significantly inhibited cell migration induced by CXCL12 in all sensitive lymphoid cells (P < 0.001). In the two less sensitive cell lines, Granta-519 and L-1236, the drug combination did not affect cell migration (Fig. 3b).

### Citarinostat + momelotinib combination induced sub-G0/G1 arrest in malignant hematological cells

**Fig. 4:**
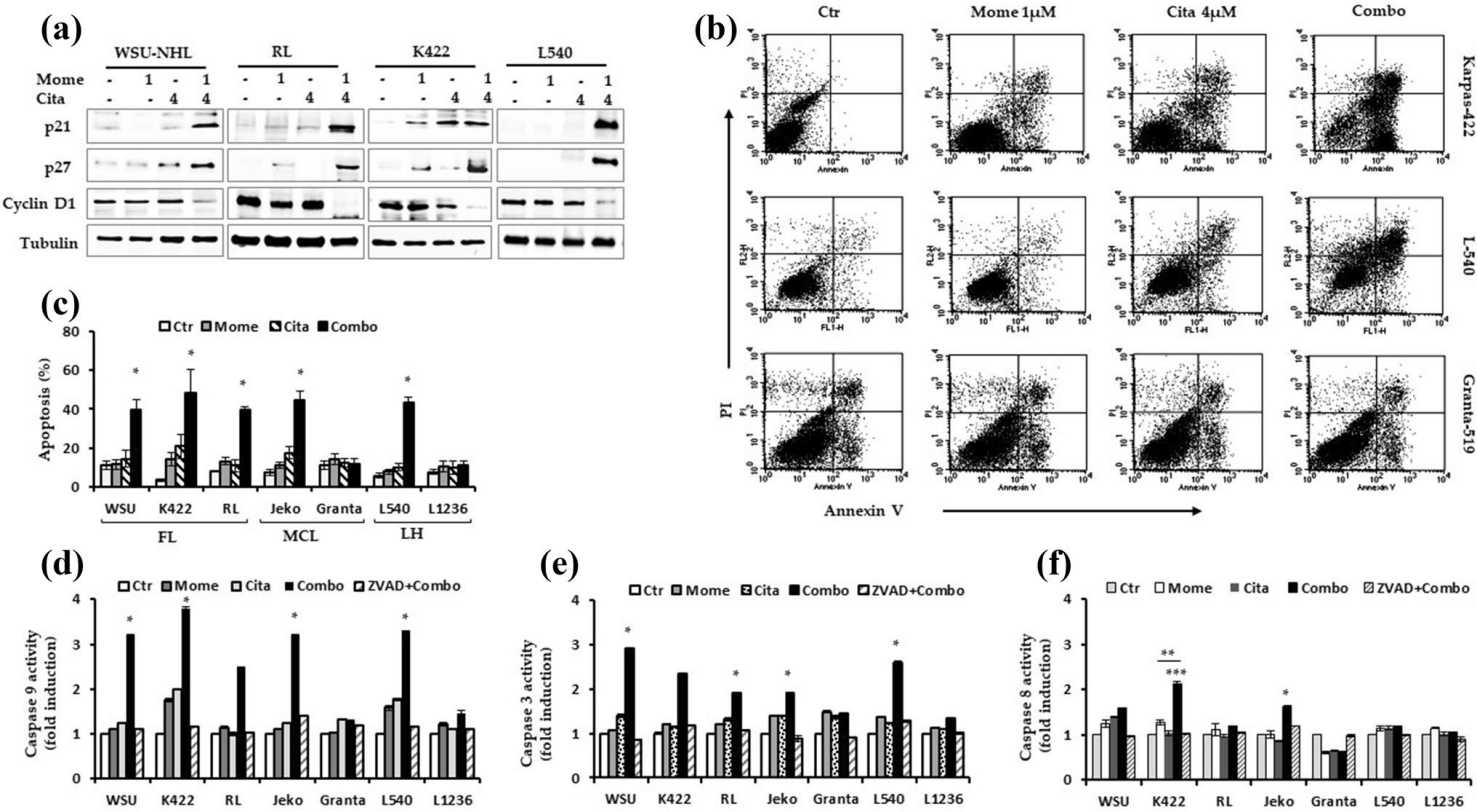
**a** Representative western blots of cellular extracts from WSU-NHL, RL, Karpas422, and L540 treated with the drugs alone and in combination for 24 h. Drug combination caused an increase of p21 and p27 proteins and in parallel the level cyclin D1 decreased. Tubulin was used to normalize protein loading. **b** Representative flow dot plot are shown for Karpas-422, L540 and Granta-519 cells (top right of panel). **c** Percentages of apoptotic cells treated with citarinostat (4 µM) and momelotinib (1 µM) (right of panel) (*p < 0.001 vs Ctr and drugs alone). **d**, **e**, **f** Cells treated with drug combination were harvested after 24 h. The apoptosis was associated with activation of caspases-9 **d** and -3 **e** in sensitive lymphoid cells. **f** The extrinsic apoptotic pathway was active only in Karpas422 and Jeko1 cells as evaluated by upregulation of caspase-8 but not in WSU-NHL, RL and L540. (* p < 0.001 vs drugs alone; ** p = 0.0040 vs momelotinib; *** p = 0.017 vs citarinostat)

In WSU-NHL, Karpas-422, RL, and Jeko-1 cells, citarinostat (4 µM) alone induced a slight increase in the percentage of cells that entered the G0/G1 phase, compared to untreated cells. In the remaining cell lines, 24-h treatments with either of the individual drugs did not significantly affect the cell cycle. In response to the citarinostat + momelotinib combination, Granta-519 and L-1236 cells did not go into cell cycle arrest. In contrast, in all sensitive cell lines, the drug combination reduced the proportion of cells in the G0/G1 and S phases. Moreover, after 24 h, the combination treatment increased the proportion of cells in the “sub-G0/G1” phase, which indicated cell death (Fig. 3c-d). We confirmed the effects of the drug combination on cell cycle-regulating proteins with immunoblotting. Treatments with the single agents did not change the expression of proteins known to play crucial roles in the cell cycle. However, the drug combination upregulated the expression of p21 and p27 proteins and downregulated cyclin D1 in four sensitive lymphoid cell lines: WSU-NHL, RL, Karpas-422, and L-540 (Fig. 4a).

### The mitochondrial apoptotic pathway and bcl-2 family proteins mediate apoptosis induced by the drug combination

We next determined whether the drug combination inhibited cell viability and cell cycle arrest in sub-G0/G1 phase by altering apoptosis. To that end, we evaluated apoptosis in both the sensitive (WSU-NHL, RL, Karpas-422, Jeko1, and L-540) and resistant (Granta-519 and L-1236) cell lines.

After 24 h, all sensitive cells showed increased apoptosis (39 to 48%, Fig. 4b-c), compared to the apoptosis observed with single agents. Minimal apoptosis was observed in Granta-519 and L-1236 cells (12% and 11.18%, respectively). The apoptosis induced by the drug combination was exerted mainly via the mitochondrial apoptotic pathway (intrinsic apoptotic pathway), as demonstrated by the upregulation of caspase-9 expression. In particular, WSU-NHL, Karpas-422, Jeko-1, and L-540 cells showed between 3.2- and 3.8-fold increases in caspase-9 expression compared to controls (Fig. 4d). This result was confirmed by applying the pan-caspase inhibitor, z-VAD-fmk. The addition of z-ZVAD inhibited the effect of the drug combination on caspase 9 in sensitive cell lines. Thus, drug-induced apoptosis was associated with caspase-3 activation, which was prevented by z-ZVAD inhibition (Fig. 4e).

**Fig. 5:**
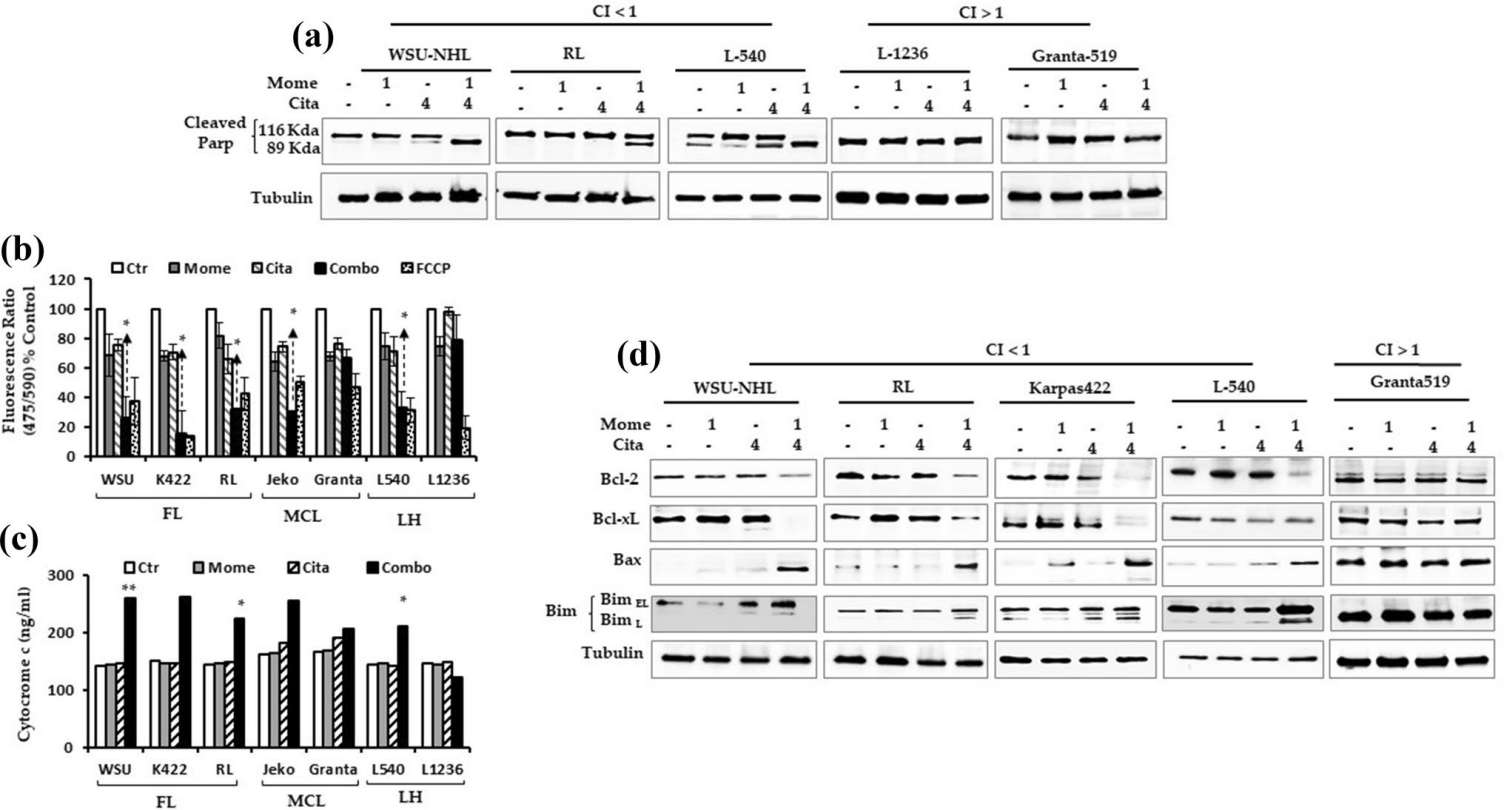
**a** Cell death induced by drugs in combination was linked to the cleavage of PARP in sensitive lymphoid cells but not in Granta-519 and L-1236 (CI > 1). Cells were treated for 24 h and whole-cell extracts were prepared and analyzed by western blot analysis using antibody against cleaved PARP. Tubulin was used to normalize the protein load. **b** Lymphoid cells were treated with citatinostat (4 µM) and momelotinib (1 µM) alone and in combination for 24 h. The percentage of cells displaying loss of mitochondrial membrane potential was analyzed by JC-1 assay. **c** Drug combination exhibited a strong cytotoxicity, evidenced by Cyt-C release of mitochondria. **d** Analysis of Bcl-2 family proteins via western blot analysis shows a clear effect in sensitive cells compared to the Granta-519 cells. Representative western blots of cellular extracts from WSU-NHL, RL, Karpas422, L540 and Granta-519 cells treated with the drugs alone and in combination for 24 h. Whole-cell lysates were subjected to Western blotting using the indicated Abs. Tubulin was used to normalize protein loading

The extrinsic apoptotic pathway was active only in Karpas-422 and Jeko-1 cells, as demonstrated by the upregulation of caspase-8 expression (Fig. 4f). In contrast, the extrinsic pathway was not active in WSU-NHL, RL, or L-540 cells. However, in all sensitive cells, the drug combination was highly cytotoxic, as evidenced by PARP cleavage (Fig. 5a), the hallmark of apoptosis. We further evaluated the role of the intrinsic mitochondrial pathway in apoptosis by examining three hallmarks of mitochondrial cell death: a change in the MMP (Δψm), the release of cytochrome c from mitochondria, and modulations in the Bcl-2 family. The loss of MMP is an early step in the intrinsic apoptosis cascade [32]; it indicates the collapse of mitochondrial integrity, which can lead to cell death. After 24 h of the combination treatment, the MMP was significantly disrupted (P < 0.001), which resulted in a 70% loss of MMP. In contrast, only 19 − 33% of the MMP was lost with citarinostat or momelotinib alone (Fig. 5b). The combination treatment caused only minimal MMP loss in Granta-519 and L-1236 cells. Additionally, the combination treatment caused elevations in cytochrome c levels in sensitive cells (Fig. 5c), but not in Granta-519 and L-1236 cells. Moreover, in sensitive cell lines, compared to single agent treatments, the drug combination reduced the levels of Bcl-2 and Bcl-xL proteins and increased the levels of pro-apoptotic Bcl-2 family members, Bax and Bim (Fig. 5d). Comparable effects were observed in L-1236 cells (data not shown), but these effects were not observed in Granta-519 cells (Fig. 5d).

### Citarinostat + momelotinib triggers ROS generation and promotes ER stress

**Fig. 6:**
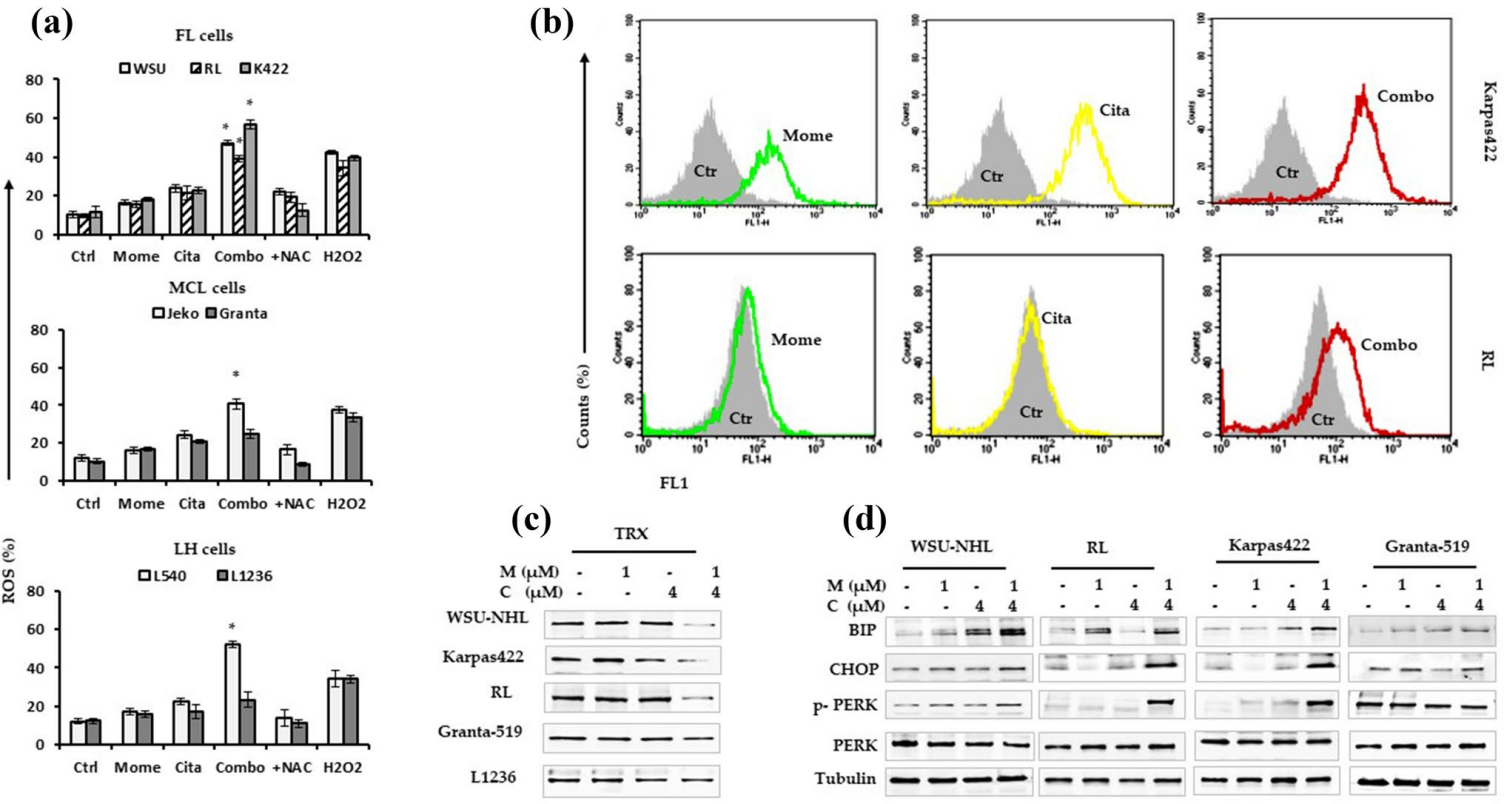
**a** Percentage of cells with increased ROS level from drug combination compared with the control cells. The co-administration of the antioxidant NAC blocked the increased of ROS generation. H_2_O_2_ was used as a positive control. Data are expressed as the mean ± SD of triplicate culture (*p < 0.001 vs drug alone). **b** Representative histograms showing ROS level from Karpas-422 and RL cells after treatment with drugs alone and in combination for 24. **c** Trx protein levels in WSU-NHL, RL, Karpas422, Granta-519 and L-1236 cells treated with the drugs alone and in combination for 24 h. **d** The induction of apoptosis was correlated with an increased expression of ER stress sensor (BIP, CHOP, PERK). Representative western blots of cellular extracts from WSU-NHL, RL, Karpas422, and Granta-519 cells treated with the drugs alone and in combination for 24 h

To clarify the mechanism of cell death induced by the combination treatment, we measured the levels of intrinsic oxidative stress in selected lymphoid cell lines. The HDACis, including citarinostat, act by stimulating ROS production [33] and activating caspases [3]. ROS production leads to caspase activation, which induces apoptosis through the extrinsic or intrinsic pathway [34]. Treating cells with single agents caused only slight effects on ROS production in selected lymphoid cells (Fig. 6a-b). In Granta-519 and L-1236 cells, the drug combination caused a modest increase in ROS production, compared to controls and compared to treatments with the individual drugs alone. In all other sensitive cell lines, the combination treatment induced a large increase in the proportion of ROS-positive cells (from 39 to 56%); we observed 3.6- to 4.8-fold increases compared to control or the single drugs alone (Fig. 6a). When lymphoid cells were co-treated with the antioxidant, NAC, the ROS levels decreased significantly (Fig. 6a-b). These results showed that ROS production played a role in the apoptosis induced by the combination treatment.

Next, we investigated whether thioredoxin (Trx), a class of cellular antioxidant proteins, played a role in protecting lymphoid cells against high levels of intrinsic oxidative stress. We compared Trx expression levels between sensitive and resistant (Granta-519, L-1236) cells. The Trx antioxidant system is integral to maintaining the intracellular redox state, which inhibits spontaneous and drug-induced apoptosis [35]. A western blot analysis indicated that exposure to the drug combination reduced Trx activity in Karpas-422, WSU-NHL, and RL cells (Fig. 6c). No significant effect was observed with the single drugs alone. Similar results were observed in Jeko-1 and L-540 cells (data not shown). In Granta-519 and L-1236 cells, the drug combination did not inhibit Trx expression (Fig. 6c). Thus, elevated Trx expression levels might be responsible for reducing the drug sensitivity of lymphoid cells.

Endoplasmic reticulum (ER) stress plays a critical role in apoptosis. In the ER, the unfolded protein response (UPR) is a stress-induced signaling pathway activated by the accumulation of unfolded proteins [36]. To determine whether the combination treatment regulated ER stress to induce cell death in lymphoid cells, we measured the status of some UPR hallmarks. We found that the drug combination upregulated the expression of hallmark ER stress proteins, BIP, CHOP, and PERK (Fig. 6d). These results indicated that the combination treatment induced apoptosis in sensitive lymphoid cells and that the induced apoptosis was correlated to changes in ER stress.

### The combination treatment downregulates mitochondrial ATP production and reduces lactate levels

**Fig. 7:**
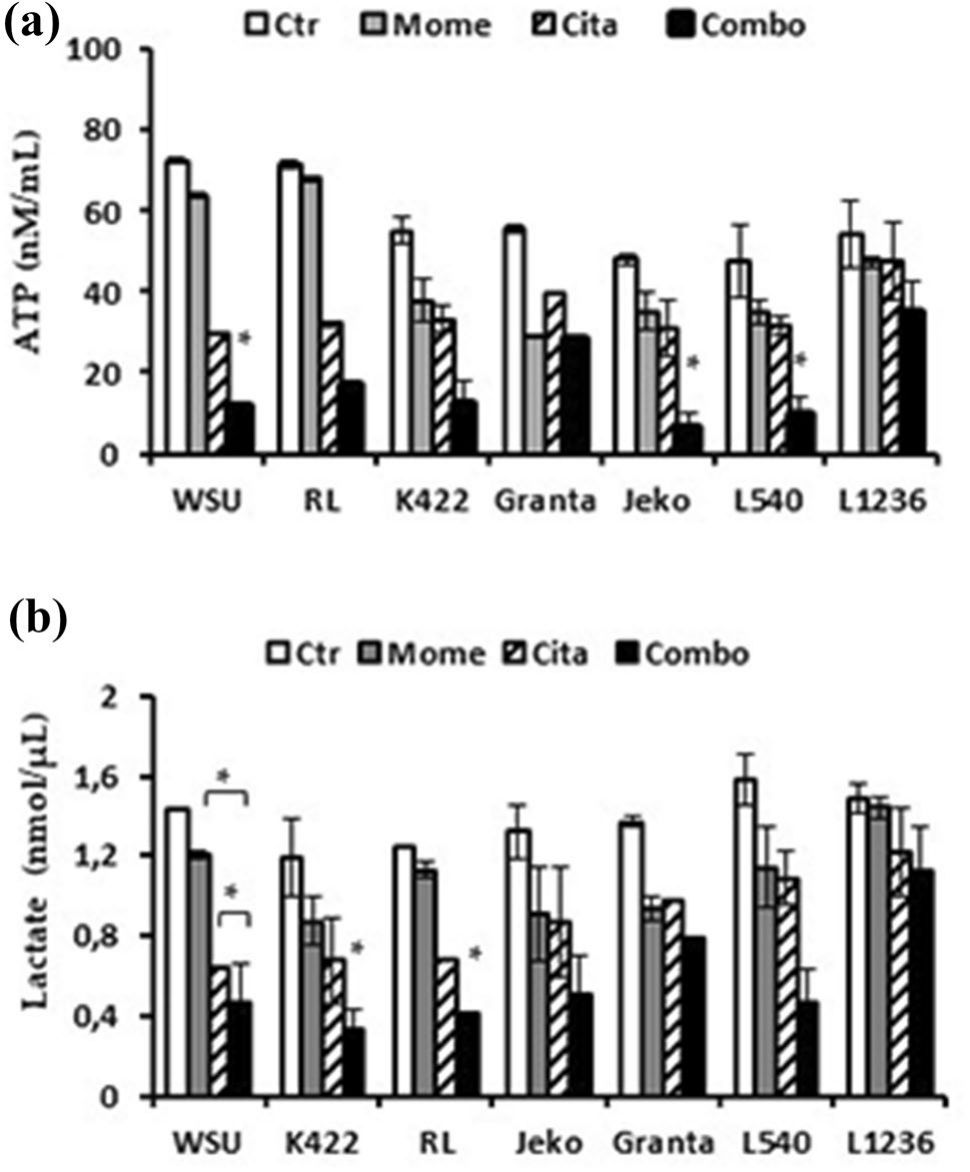
**a** Intracellular ATP levels after 24 h with drugs alone and in combination. Data are expressed as the mean ± SD of triplicate culture. **b** Lactate levels after treatment with drugs alone and in combination were determined in the supernatant by colorimetric analysis. Data are expressed as the mean ± SD of triplicate culture (*p < 0.001 vs drug alone)

Mitochondria play central roles in cell viability. They produce ATP to provide energy and they induce apoptosis to cause cell death [37]. The hallmarks of cell apoptosis are preceded by mitochondrial alterations, including a loss of MMP and a reduction in ATP production. Therefore, we measured the ATP content in sensitive cell lines after 24-h exposures to 4 µM citarinostat combined with 1 µM momelotinib. Figure 7a shows that the combination treatment downregulated mitochondrial ATP production (P < 0.05). Next, we measured lactate consumption in sensitive cells after the combination treatment. We found that lactate levels were reduced by 33%, compared to the respective controls (p < 0.01). In contrast, the Granta-519 and L-1236 cell lines showed no significant differences in ATP content or lactate consumption (Fig. 7b). These data indicated that the combination drug treatment probably induced ROS formation by causing mitochondrial damage. This damage caused in a decline in ATP content and a reduction in the glycolytic rate, which resulted in cell death.

### The combination treatment was accompanied by JAK and STAT dephosphorylation and α-tubulin acetylation

**Fig. 8:**
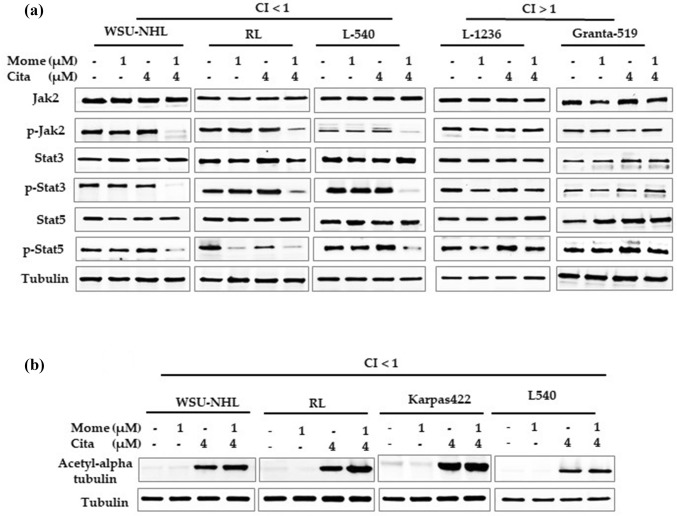
**a** Drug combination induced inactivation of the JAK2 / STAT3 pathway and its downstream mediators, including STAT3 and STAT5 in the sensitive lines with respect to the Granta-519 cell line. Whole-cell lysates were subjected to western blotting using the indicated Abs. Tubulin was used to normalize the protein load. **b** The exposure of citarinostat induced the acetylation of α-tubulin in lymphoid cells, the extent of which was not further modified by momelotinib. Tubulin was used to normalize the protein load

Momelotinib therapy targets the JAK/STAT family, a key prosurvival pathway that controls apoptotic signals in hematological malignancies [38]. To evaluate this effect, we treated sensitive lymphoid cells with the citarinostat + momelotinib combination for 24 h, and we collected cell lysates for western blotting. The expression levels of p-JAK2, p-STAT-3, and p-STAT-5 proteins were lowered compared to the untreated control group. Similar results were observed in Jeko-1 cells (data not shown), but Granta-519 and L-1236 cells showed no changes in JAK/STAT expression levels (Fig. 8a). Previous studies have shown that HDAC6 actively controlled α-tubulin acetylation and that citarinostat treatment caused α-tubulin hyperacetylation [6, 39]. We found that, in sensitive lymphoid cells, citarinostat alone induced α-tubulin acetylation (Fig. 8B), and this activity was not further modified by adding momelotinib (Fig. 8b). We obtained similar results in Jeko-1 cells (data not shown).

### Trx silencing in resistant lymphoid cell lines sensitized cells to the drug combination by reducing Bcl-2 expression and activating caspase 3

The drug combination had limited effects on cell viability, cytotoxicity, apoptosis, and ROS generation in the Granta-519 and L-1236 cell lines, with CIs > 1. In the present study, we found that Trx expression levels were different between sensitive and resistant lymphoid cells. It is well known that Trx plays a role in protecting cells against stress-induced cell death [40] by preventing oxidative stress and maintaining the cellular redox environment. Consequently, we hypothesized that Trx might play a role in the resistance of lymphoid cells to the drug combination.

**Fig. 9:**
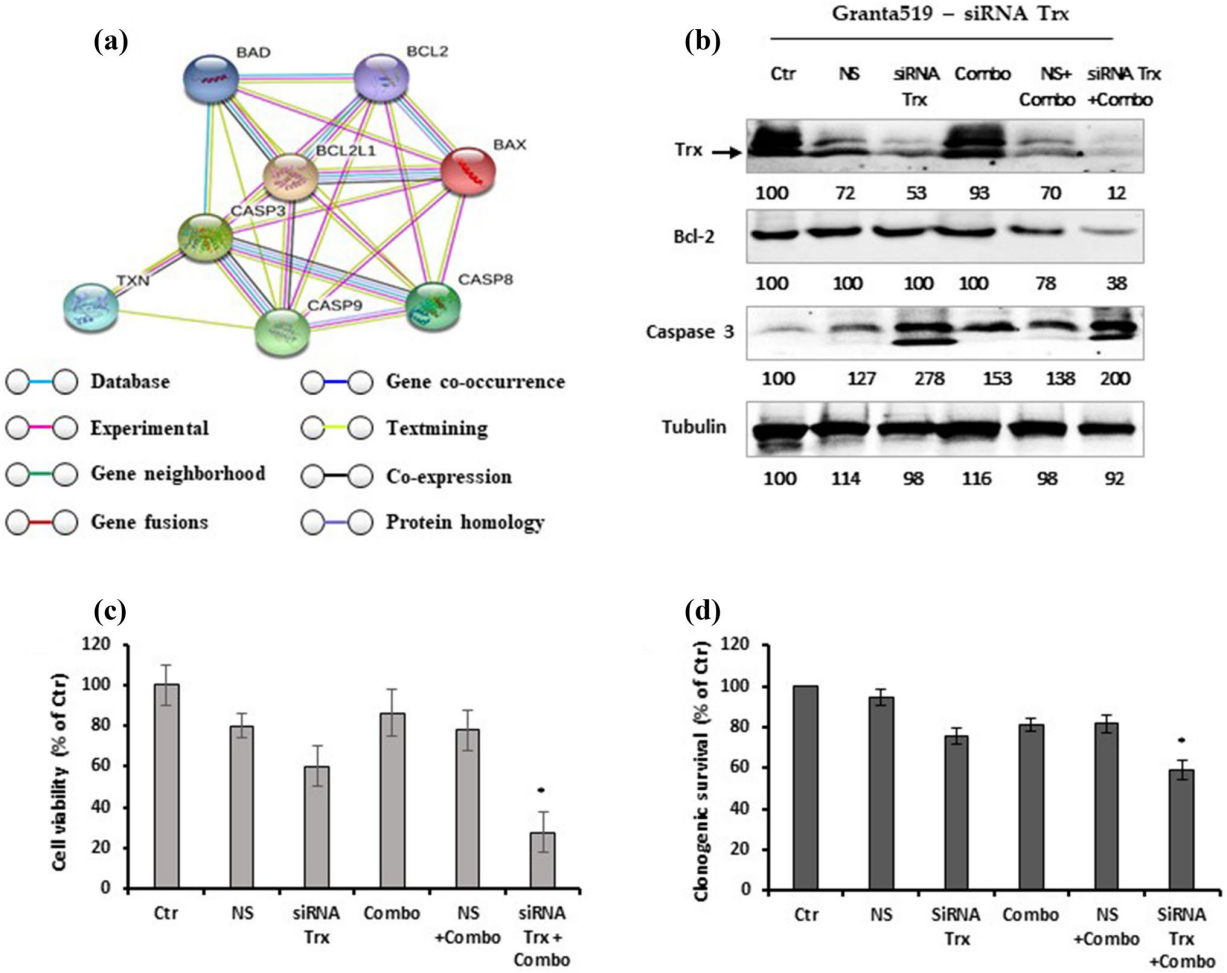
**a** Genetic interaction network associated with thioredoxin. In this network, circle represent node (proteins), while line represents edge (connections). **b** Effect of thioredoxin siRNA in Granta-519. Inhibition of thioredoxin decreases Bcl-2 and activates caspase 3. A representative western blot of Trx protein at 48 h after transfection. Whole-cell lysates were subjected to western blotting using the indicated Abs. Densiometric semi-quantification of bands normalized to the control is shown below the immunoblot bands. Tubulin was used to normalize the protein load. **c** Transfection of siRNA-thioredoxin reduced Granta-519 cell viability. Cell viability was measured at 48 h after transfection. **d** siRNA-Trx reduced clonogenic survival of Granta-519 cells. The cells were seeded onto methylcellulose media for cloning six hours after transfection. Data represent percentage of colonies of control siRNA-transfected cell of three independent experiment. (*p < 0.001)

We also explored molecular interactions between Trx and other proteins involved in cell cytotoxicity. We employed String (https://string-db.org/) to establish an in silico genetic interaction network for predicting associations between Trx and apoptosis. This network analysis predicted several key hubs associated with apoptosis and Trx, according to a literature search. Figure 9a shows the predicted key proteins, including Bcl-2 and caspase 3, and their relationships to apoptosis. We used siRNAs to knock down Trx in Granta-519 cells and investigated whether these cells were sensitized to the combination treatment. We performed western blot analyses to test our hypothesis. The results indicated that knocking down Trx in Granta-519 cells reduced Trx expression levels, reduced the levels of the anti-apoptotic protein Bcl-2, and activated caspase 3 (Fig. 9b). These effects reduced the resistance of Granta-519 cells to treatment. Upon exposure to the combination treatment, Trx-silenced Granta-519 cells showed reduced cell viability and significantly reduced clonogenic survival (Fig. 9c-d). We hypothesized that the Trx deficiency in resistant cells contributed to the activation of cytotoxicity, growth arrest, and the loss of clonogenicity.

## Discussion

Many lymphoid malignancies are associated with increased HDAC expression and activity [10–12, 41]. HDACs are key epigenetic regulators and promising therapeutic targets for cancer treatment. HDACis represent an encouraging class of antitumor drugs; they induce a series of molecular and biological responses with minimal toxicity to normal cells. Recently, attention has been focused on the development of selective HDACis, based on the principle that selective agents might be more tolerable than pan-HDACis. HDAC6 has emerged as a target for the potential selective inhibitors studied to date [9]. Among the selective HDACis in development, only ricolinostat and citarinostat have been evaluated in clinical trials [16]. Citarinostat was designed as a second-generation, orally available drug, with HDAC6 selectivity. Its solubility properties are improved over the structurally related inhibitor, ricolinostat [17]. In our previous “in vitro” preclinical study [6], we demonstrated synergy between ricolinostat (Acy-1215) and bendamustine, which strengthened the rationale for using citarinostat (ACY-241) in combination with other drugs. The main benefits of using combined therapies are that lower doses can be used with better tolerability and the development of drug resistance can be prevented or delayed. Thus, combining drugs has become an attractive strategy for cancer treatments.

Dysregulated JAK-STAT signaling leads to increased proliferation and/or decreased apoptosis in neoplastic cells. Momelotinib is a potent inhibitor of JAK1 and JAK2. Momelotinib has been studied in myeloproliferative diseases, myeloma, and solid cancer [16, 25–27]. We reasoned that inhibiting both HDAC and JAK/STAT activities with citarinostat + momelotinib might represent a promising novel therapeutic modality for lymphoid malignancies. In the present study, we tested this drug combination in different lymphoid cell lines. We observed that the tested cell lines had different sensitivities to the single agents. The drug combination provided greater efficacy in all cell lines, except two lines, Granta-519 and L-1236, which showed little sensitivity to the combination treatment. In the sensitive cell lines (WSU-NHL, RL, Karpas-422, Jeko-1, and L540) the citarinostat + momelotinib combination induced apoptosis mediated by the intrinsic (mitochondrial) apoptotic pathway. The apoptosis was followed by caspase activation, which triggered programmed cell death. The activated caspases were inhibited by z-VAD, a pan-caspase inhibitor, which indicated that the drug combination induced apoptotic cell death associated with caspase activation. However, not all the tested cell lines showed the same sensitivity to treatment in terms of caspase activation. Moreover, 24-h exposures to either drug alone did not induce caspase activation or pro-apoptotic factor release into the cytosol. The cleavage of caspase 8 was detected in only two cell lines (Karpas-422 and Jeko-1), which suggested that the extrinsic apoptotic pathway was activated in some cells. With the exception of Granta-519 and L-1236 cells, which did not appear to be very sensitive to the combination treatment, all of the examined lymphoid lines displayed caspase-3 and -9 cleavage and PARP activation. Thus, the combination treatment activated mitochondrial events and induced the release of pro-apoptotic factors.

The disruption of the mitochondrial membrane is considered the “point of no return” for apoptotic cell death. This disruption causes the release of cytochrome c and other pro-apoptotic factors into the cytoplasm [50]. The present study showed that the combination treatment disrupted the mitochondrial membrane, which caused cytochrome c release and ROS generation. Tumor cells are characterized by a high proliferation rate, a high metabolism, and consequently, elevated ROS levels [42, 43]. Mitochondria are also a major source of ROS generation. Elevated ROS can increase cellular oxidative stress and cause irreversible damage to cellular lipids, DNA, and/or proteins, which can directly induce cancer cell death [44]. Interestingly, pretreating the lymphoid cells with NAC inhibited the apoptosis mediated by the combination treatment. This result suggested that ROS might play a substantial role in the anticancer activity of this drug combination. Oxidative stress is mainly generated in the mitochondria [42], and mitochondria play a central role in cell survival and growth [32]. Mitochondrial-induced apoptotic events are controlled and regulated by members of the Bcl-2 family proteins [46]. These proteins regulate mitochondrial membrane permeability and have either pro-apoptotic (e.g., Bax and Bim) or anti-apoptotic (e.g., Bcl-2 and Bcl-xL) activities [47]. It is thought that the main mechanism of action of Bcl-2 family proteins is to regulate cytochrome c release from the mitochondria by altering mitochondrial membrane permeability [47]. We found that the combination treatment increased the expression of pro-apoptotic proteins, Bax and Bim, and reduced the levels of anti-apoptotic proteins, Bcl-2 and Bcl-xL. Elevated ROS levels and perturbations in intracellular redox homeostasis induce the accumulation of unfolded proteins in the ER, which triggers the ER stress response [48]. Prolonged or severe ER stress can lead to apoptosis. ER-stress is associated with the induction of high expression levels of the C/EBP homologous protein, CHOP. CHOP is a transcription factor that is thought to play a key role in ER-stress-mediated apoptosis [49]. In the present study, a western blot analysis showed that the drug combination-induced apoptosis occurred through the ER stress pathway. We observed increases in three hallmarks of ER stress: CHOP, BIP, and PERK. Oxidative stress can also induce tumor cell death indirectly by modulating many redox-dependent cellular pathways that mediate cell survival [45]. To counterbalance oxidative stress, tumor cells up-regulate antioxidant proteins, such as Trx [50–52]. Trx is a small, inflammation-inducible, oxidoreductase protein that is ubiquitously expressed in all organisms. Trx is involved in a wide range of physiological cellular responses, both inside and outside the cell [35]. Inside the cell, Trx alleviates oxidative stress by scavenging ROS, which produces cytoprotective effects. In transformed lymphoid cells [53, 54], Trx acts as a growth factor that stimulates cancer cell proliferation and tumor growth and inhibits spontaneous and drug-induced apoptosis [35]. Trx overexpression caused aggressive tumor growth, inhibited apoptosis, and was correlated with reduced patient survival [35, 55, 56] and a poor prognosis [56]. Trx was also correlated with chemotherapy resistance in various human hematologic malignancies, including B-cell lymphoma [57]. In cancer cells, Trx inhibition increased the sensitivity to cell death [58]. Our results showed that Trx expression levels were different in drug-sensitive and drug-resistant lymphoid cells. The combination treatment reduced Trx levels in sensitive lymphoid cells, but did not affect Trx levels in Granta-519 or L-1236 cells. When we knocked down Trx with siRNA in vitro, the combination treatment- induced apoptosis in treatment-resistant Granta-519 cells, shown by the reduction in Bcl-2 expression and the activation of caspase 3. Trx-deficient Granta-519 cells displayed reductions in cell viability and clonogenic survival. Therefore, Trx might serve as a potential drug target for overcoming drug resistance in relapsed/refractory lymphoid cells. Thus, targeting Trx could have therapeutic effects.

In conclusion, the present study demonstrated that citarinostat combined with momelotinib had anticancer effects in hematological malignant cell lines in vitro. Our results suggested that this combination treatment sensitized lymphoid cells to multiple cell death mechanisms, including mitochondrial apoptosis pathways; elevated ROS production, followed by ER stress; and cell cycle perturbation through JAK and STAT3 signaling modulation. Due to their favorable toxicity profiles and the oral formulations, the citarinostat + momelotinib combination showed promise as a novel therapeutic modality for hematological malignancies. However, not all tested cell lines showed equivalent treatment sensitivity. Future studies should aim to overcome resistance mechanisms.

## Electronic supplementary material

Below is the link to the electronic supplementary material.

Supplementary file1 (JPG 159 kb)

Supplementary file2 (JPG 157 kb)

## Abbreviations

ANOVA: Analysis of variance
BM: Bone marrow
BSA: Bovine serum albumin
Cis: Combination indices
CXCL12: C-X-C Motif Chemokine Ligand 12
DCFH-DA: 5 μM 2′,7′-dichloroflourescein diacetate
DMEM: Dulbecco's modified Eagle’s medium
ER: Endoplasmic reticulum
FBS: Fetal bovine serum
FCCP: Carbonyl cyanide 4-(trifluoromethoxy) phenylhydrazone
FITC: Fluorescein isothiocyanate
GFP: Green Fluorescent Protein
HDACis: Histone deacetylases inhibitors
HDACs: Histone deacetylases
hMSCs: Human mesenchymal stem cells
MMP: Mitochondrial membrane potential
MTS: (3-(4,5-Dimethylthiazol-2-yl)-5-(3-carboxymethoxyphenyl)-2-(4-sulfophenyl)-2H-tetrazolium)
NAC: N-acetyl-l-cysteine
PARP: Poli ADP-ribosio polimerasi
PBMCs: Peripheral blood mononuclear cells
PBS: Phosphate-buffered saline
ROS: Reactive oxygen species
RPMI: Roswell Park Memorial Institute
SDF-1α: Stromal cell-derived factor 1
siRNAs: Small interfering RNAs
Trx: Thioredoxin
UPR: Unfolded protein response
zVAD-fmk: Carbobenzoxy-valyl-alanyl-aspartyl-[O-methyl]- fluoromethylketone
