# Supplementary figures and images for: Citarinostat and Momelotinib co-target HDAC6 and JAK2/STAT3 in lymphoid malignant cell lines: a potential new therapeutic combination
Source: Apoptosis. 2020 May 11;25(5):370–87. doi: 10.1007/s10495-020-01607-3 (PMC7244621; doi:10.1007/s10495-020-01607-3)

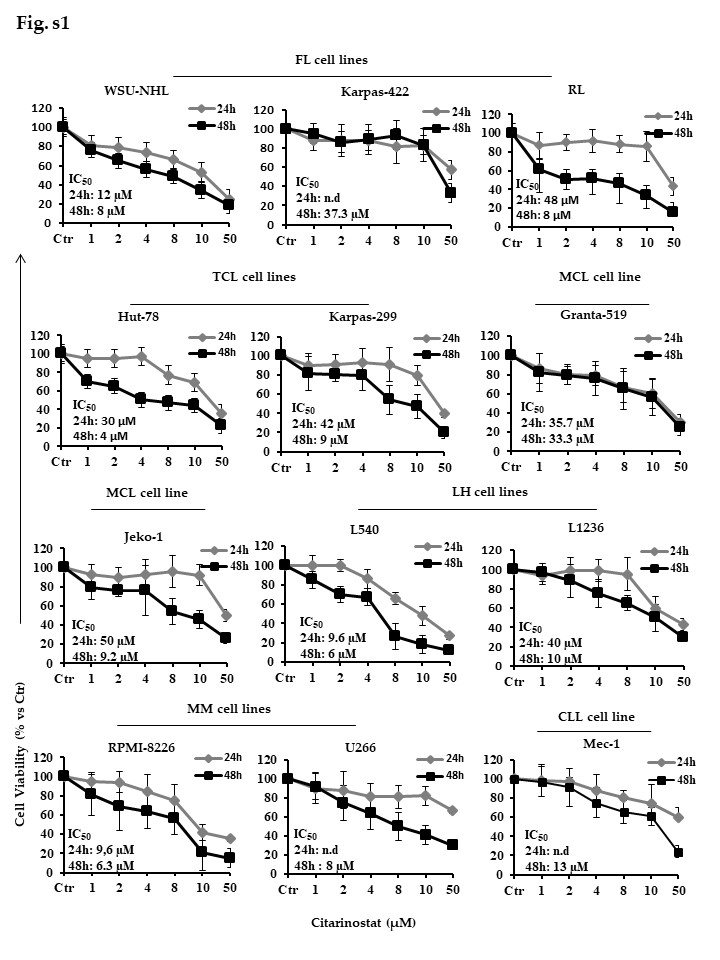

Supplement: Supplementary file 1 — Supplementary file1 (JPG 159 kb) [file 10495_2020_1607_MOESM1_ESM.jpg]

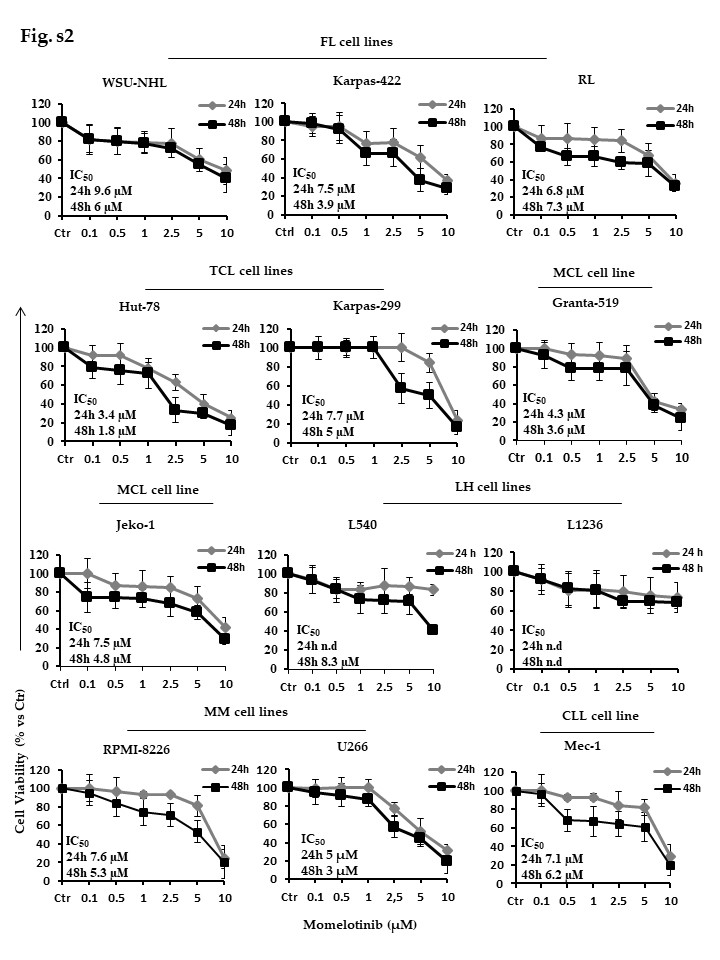

Supplement: Supplementary file 2 — Supplementary file2 (JPG 157 kb) [file 10495_2020_1607_MOESM2_ESM.jpg]
